# Incidental Adrenal Mass in a Patient With a Known History of Neurofibromatosis: A Case Report

**DOI:** 10.7759/cureus.93008

**Published:** 2025-09-23

**Authors:** Torrin P Jacobsen, Gizem B Keles, Sara S Amer, Valerie Mogilevskiy, Kamal T Patel

**Affiliations:** 1 Pain Management, Lake Erie College of Osteopathic Medicine, Tampa, USA; 2 Pain Management, Tampa Bay Pain Associates, Tampa, USA

**Keywords:** acute low back pain, adrenal pheochromocytoma, chronic low back pain, cutaneous neurofibromas, incidental adrenal mass, interventional pain management, neurofibromatosis 1, nf1, pheochromocytoma, sympathetic paraganglioma

## Abstract

Neurofibromatosis type 1 (NF1), formerly known as von Recklinghausen disease, is the most common clinical phenotype of neurofibromatosis. Autosomal dominant variants in the NF1 gene, located on chromosome 17q11.2, lead to diverse clinical manifestations, including café-au-lait macules, cutaneous neurofibromas, Lisch nodules, optic gliomas, seizures, pheochromocytomas, and osseous lesions. We present a case report on a 63-year-old female with NF1 who presented with chronic radicular back pain that had recently changed in character and distribution.

A computed tomography (CT) scan ordered for the evaluation of the patient's altered pain symptoms incidentally revealed a left adrenal mass measuring 4.1 x 3.1 cm. CT imaging was selected due to its superior spatial resolution, essential for assessing bony abnormalities associated with chronic spinal conditions. Although normotensive and without classic symptoms, such as tremors or headaches, the patient reported episodic heat intolerance with feelings of overheating. Given her medical history, physical exam findings, and imaging results, pheochromocytoma or sympathetic paraganglioma was strongly suspected. Endocrinology referral was advised; however, the patient did not complete this consultation at the time of this report. Consequently, the diagnosis of pheochromocytoma remains presumptive.

This case underscores the critical role incidental imaging findings play in the early identification and management of pheochromocytomas and sympathetic paragangliomas, especially in NF1 patients. It emphasizes the necessity for heightened clinical vigilance and interdisciplinary follow-up in atypical presentations lacking classic symptomatology.

## Introduction

Neurofibromatosis type 1 (NF1) is estimated to have a prevalence of 1:2,052, with an estimated incidence between 1:2,558 and 1:3,333 births [1]. NF1 carries a decreased life expectancy of about 15 years, with malignancy of connective and other soft tissue being reported as the leading cause of death [2]. Symptoms vary in severity, with Lisch nodules (>95%), cutaneous neurofibromas (99%), café-au-lait macules (>99%), optic gliomas (15-20%), and pheochromocytomas and paragangliomas (<1%) being present in individuals with the disease [1]. Pheochromocytomas are rare neuroendocrine tumors occurring in 2-8 per million people in the general population, but develop in almost 6% of patients affected by NF1 in their lifetime [3,4]. Because of the rarity of pheochromocytoma among the general population and its increased incidence among those diagnosed with NF1, the relationship between symptomatology, imaging findings, and patient presentations in any specialty setting is important. 

Pheochromocytoma symptoms consist of episodic hypertension and a classic triad of paroxysmal headache, palpitations, and diaphoresis. In addition, anxiety, pallor, dyspnea, and orthostatic hypotension have been noted [1,5,6]. Clinical diagnostic results include elevated metanephrines more than twice the upper limit of normal [1,4]. Common imaging modalities are computed tomography (CT) of the abdomen and pelvis, which is considered first line for imaging. CT imaging is preferred over MRI due to the spatial resolution [1,6,7]. Incidental imaging is a common method of detection in pheochromocytoma, with 23-58% of pheochromocytomas being found incidentally on imaging [7-9].

Paragangliomas are also considered rare neuroendocrine tumors arising from extra-adrenal autonomic paraganglia. They are closely related to pheochromocytomas and are indistinguishable at the cellular level and may even present similarly with hypertension, episodic headaches, sweating, and tachycardia [3,4]. However, within the realm of NF1, pheochromocytomas are much more frequent than paragangliomas [3,4].

Here, we present the case of a 63-year-old female with a known history of NF1 who experienced a change in her chronic radicular back pain symptoms, prompting further evaluation. Imaging ultimately revealed an incidental adrenal mass. This case is particularly relevant in the context of pain management, as the patient’s existing medication regimen, which had been developed for chronic pain, required review given the possibility of a catecholamine-secreting tumor. There is limited literature addressing safe analgesic choices and chronic pain management strategies in patients with pheochromocytomas or paragangliomas, making this case valuable for both diagnostic and therapeutic considerations [10].

## Case presentation

A 63-year-old female with a known history of NF1 and longstanding chronic radicular back pain presented to an interventional pain clinic due to changes in symptoms associated with her pain. She previously had chronic low back pain associated with lumbar facet joint arthropathy and lumbar spinal stenosis, leading to radicular symptoms along the L5-S1 roots. These previous symptoms were managed symptomatically. She had previously undergone two spinal epidural injections over the course of one year, and one round of lumbar paraspinal radiofrequency ablations (RFAs). Pharmacologic therapy was initiated with methocarbamol 500 mg twice daily and eventually increased to 750 mg four times daily. However, oxycodone 10 mg orally 3 times daily was added due to inadequate symptom relief. As her radicular symptoms progressed, pregabalin, targeting neuropathic pain, was added and titrated to 250 mg orally three times daily. She reported adequate symptomatic control on this combination regimen for the past five months, prior to the onset of new symptoms.

The patient stated that her symptoms felt higher than her normal, but she didn’t initially attribute much significance to this. Upon further questioning, she reported the onset of new bilateral mid-to-lower back pain, worse on the left, radiating into her left lower extremity over the past several months. Pain was characterized as constant, deep, throbbing, cramping, and shooting in nature. She also mentioned episodes of overheating that began shortly before the new-onset symptoms of back pain. The patient denied palpitations or headaches. The patient also denied any urinary and/or bowel incontinence. Questioning revealed the patient, along with her sister, had been diagnosed with the NF1 subtype in early adolescence after evaluation for developing multiple neurofibromas over their trunks.

A thorough physical examination was conducted, including orthostatic blood pressure measurements and blood glucose levels, due to the patient’s report of episodic overheating and known NF1 association with pheochromocytomas. Orthostatic vital signs, temperature, and fasting blood glucose levels were within normal limits (Table 1). Physical examination revealed diffuse tenderness over the mid-back region without discrete localized areas. Findings unchanged from previous examinations included numerous cutaneous neurofibromas covering her face and body, café-au-lait macules, and bilateral Lisch nodules.

**Table 1:** Patient's vitals upon basic screening for orthostatic hypotension and fasting blood glucose levels due to the known association of pheochromocytomas with hypoglycemia and orthostatic hypotension. Temperature was taken during patient intake prior to knowledge of “overheating”. bpm = beats per minute

Parameter		Value	Reference Range
Blood Pressure	Seated Blood Pressure (mmHg)	122/63	< 110/75 mmHg
	Standing Blood Pressure (mmHg)	120/60	
Pulse	Seated Pulse (bpm)	78	70-100 bpm
	Standing Pulse (bpm)	76	
Fasting Blood Glucose (mg/dL)		90	65-109 mg/dL
Temperature (ºC)		36.7	35.9-37.3ºC

A brief neurologic exam showed slightly diminished bilateral lower extremity strength (4/5 throughout), with symmetric patellar and Achilles reflexes (2+), and absent Babinski sign bilaterally. Sensation to light touch and pinprick was intact across the thoracic and lumbar dermatomes. No focal motor deficits were noted. Gait was normal. No palpable masses or lymphadenopathy were identified.

The patient was advised to consult endocrinology for thyroid evaluation due to reported overheating episodes, but had not done so by the follow-up appointment. Upon returning with CT imaging results, the radiology report indicated a left adrenal mass measuring 4.1 x 3.1 cm, which was confirmed upon review by the medical team. There was no other structural cause identified that could explain the change in symptoms with her back pain. The spine was unchanged from previous imaging conducted less than six months ago, which showed stable lumbar stenosis and lumbar facet joint arthropathy with no new changes or osteophyte formations.

Given the high suspicion for pheochromocytoma based on incidental imaging findings and clinical context, the medical team reconsidered her current medication regimen to mitigate potential complications. However, none of the patient’s medications prescribed for pain management (oxycodone, pregabalin, and methocarbamol) were altered. We discussed the avoidance of meperidine, methadone, and morphine with the patient, should she need a change in regimen in the future.

Despite the lack of classic triad symptoms in the patient, the physical exam findings, genetic history, and imaging findings allowed the physician to assign a pheochromocytoma or sympathetic paraganglioma as a differential diagnosis of the incidental adrenal mass. However, the diagnosis of pheochromocytoma or sympathetic paraganglioma was not definitively confirmed at the time of this report. The patient did not undergo surgical resection of the mass, and there is no documentation indicating completion of biochemical testing (e.g., plasma metanephrines or 24-hour urine catecholamines).

The patient was advised to obtain further diagnostic workup and reconsider endocrine evaluation; however, the patient has since moved, and no additional laboratory or imaging studies were documented. As such, the diagnosis of pheochromocytoma or sympathetic paraganglioma remains presumptive based on imaging and clinical suspicion, and treatment was not initiated through the pain clinic. No adverse or unanticipated events were reported during the course of care.

## Discussion

Autosomal dominant variants in the NF1 gene, located on chromosome 17q11.2, lead to diverse clinical manifestations, including café-au-lait macules, cutaneous neurofibromas, Lisch nodules, optic gliomas, seizures, pheochromocytomas, and osseous lesions [1].

Due to pheochromocytomas being much more frequent (95%; 14% bilateral) than paraganglioma (6%), we will be focusing on pheochromocytomas for the discussion [4].

In this case presentation, the relationship of the pain symptomatology and its possible connection with pheochromocytoma is of importance. Pain has been previously reported as a present symptom and associated with an underlying pheochromocytoma; specifically in the lower lumbar region, flank, and upper abdomen [8,9,11,12]. Reports of tumors associated with pain have ranged in size from 5 x 4 cm to 12 x 10 cm [9,11]. Though it is difficult to conclude that the pain associated with pheochromocytoma is caused by size, it is a possibility. For example, neuropathic cancer pain can be due to a sizable tumor compressing a nerve, creating fibrosis, and eliciting pain due to nerve damage [13]. Such damage can create heightened sensitization of nerve fibers for an extended period of time, even after insulting causes are removed [13]. Similar compressive pathophysiology in this case may be present. Due to the location of the adrenal gland and its relationship with nearby structures, nerve dysfunction and pain may localize to the thoracic spine and nearby musculature. The adrenal gland is innervated by type B preganglionic nerve fibers from the T5-T8 thoracic spinal levels, but some fibers of the greater splanchnic nerve bypass the celiac ganglion and directly synapse on chromaffin cells within the adrenal medulla [14]. Thus, nerve dysfunction may refer pain throughout the T5-T8 dermatomal pattern. In addition, increased catecholamines have been shown to increase the perception of pain due to the activation of mechano-insensitive nociceptors [15]. It is plausible that this may play a role in the perception of the pain experienced by our patient, which led to repeated evaluation with interventional pain, which ultimately led to the incidental discovery of the adrenal mass.

Treating pain in patients with pheochromocytoma may be difficult due to potential medication adverse effects [10,16]. Despite this, there is little to no standardization of permissible pain management medications, as most recommendations are based on case reports or smaller studies [10]. In regard to pain, medications contraindicated are those that may stimulate mast cell release of histamine, which then provokes catecholamine release from the pheochromocytoma [10]. Contraindicated pain medications that a patient can be prescribed in the outpatient setting include meperidine, methadone, morphine, and nalbuphine [10]. However, the Summary of Product Characteristics for Morphine and the British National Formulary indicate that fentanyl, hydromorphone, buprenorphine, or oxycodone are permissible [10,16]. In addition, the World Federation of Societies of Anaesthesiologists reaffirms fentanyl’s safety, along with remifentanil and alfentanil, in patients with pheochromocytoma [16].

These same principles of avoiding histamine-releasing drugs extend beyond outpatient pain management and into the perioperative setting. For example, succinylcholine, a depolarizing neuromuscular blocker, can provoke catecholamine release from pheochromocytomas through increased intra-abdominal pressure and muscle fasciculations, in addition to its histamine-releasing potential [10]. This risk is particularly important when pheochromocytoma is incidentally discovered, as patients undergoing unrelated surgical procedures or emergency airway management may be exposed to succinylcholine before this risk can be recognized. Similarly, desflurane and halothane--due to sympathetic stimulation and catecholamine sensitization, respectively, and ketamine--due to its sympathomimetic properties, should be avoided in this setting [17].

Ultimately, safe pain control in patients with known or suspected pheochromocytoma requires a multidisciplinary approach that bridges acute/chronic pain management and perioperative planning. Awareness of high-risk agents, both in the outpatient and anesthesia settings, helps prevent catecholamine surges and associated complications. This is especially important when a tumor is incidentally discovered in a patient already on long-term analgesic therapy, as medication regimens may need urgent modification to minimize risk while maintaining adequate pain relief.

While this case focuses on the diagnostic challenge and medication considerations in a patient with suspected pheochromocytoma, it is important to recognize that definitive management remains surgical [18-21]. Understanding the standard treatment pathway is crucial for clinicians, as incidental discoveries should prompt adjustments in medication regimens and timely referral for curative intervention.

In the majority of cases, adrenalectomy with preoperative medical preparation (PMP) using antihypertensive medications is the current standard of treatment for pheochromocytoma, to avoid a hypertensive crisis while manipulating the tumor [18-21]. The antihypertensive protocol includes using a non-selective α-antagonist, such as phenoxybenzamine, followed by a β-antagonist to combat the reflex tachycardia associated with non-selective α-blockade [19-20]. Other PMP methods include using a selective α-antagonist, such as doxazosin, which confers fewer side effects and is shorter-acting [19]. Some evidence exists for using calcium-channel blockers such as PMP due to their renal and cardioprotective effects, though this is not considered standard therapy [17,20]. Regardless of PMP status before surgery, the Roizen criteria from 1982 is still used to assess adequate α-blockade: no in-hospital blood pressure >160/90 mmHg for 24 hours before surgery; no orthostatic hypotension with blood pressure <80/45 mmHg, no ST or T wave changes for 1-week before surgery; and no more than 5 premature ventricular contractions per minute [20]. Surgical intervention can be in the form of laparoscopic adrenalectomy using a retroperitoneal or transabdominal approach, and the approach used is largely based on the surgeon’s experience, though both approaches come with their advantages and disadvantages [18,21]. The overall survival for pheochromocytoma and paraganglioma are 1-year, 3-year, 5-year, and 10-year rates of 87.4%, 75.3%, 66.6%, and 48.9%, respectively [22]. Distant metastasis and age of diagnosis over 60 years old have a significantly increased odds ratio of death, OR = 4.8 and 5.6, respectively [22].

Despite not having a definitive diagnosis in this case, the incidental detection of an adrenal mass in a patient with known NF1 highlights the consideration of a pheochromocytoma or sympathetic paraganglioma in the differential. This case also highlights the critical role of early interdisciplinary collaboration between pain management, anesthesia, endocrinology, and surgery to address both diagnostic uncertainty and the safety implications of ongoing medication regimens. Given the potential morbidity and mortality associated with undiagnosed catecholamine-secreting tumors, prompt recognition, risk mitigation, and appropriate referral remain essential, regardless of whether the diagnosis is confirmed or still under investigation.

Limitations

The limitations of this report consist of the practice setting in which the incidental finding happened, with no definitive diagnosis being made. Being a specialty clinic, the medical management and follow-up of the patient were difficult due to limited involvement as compared to a primary care provider. In addition, the imaging was not available to add to this case report. The limited literature on pheochromocytoma and its relationship with pain leaves the associations of pain as plausible versus explicitly causative.

## Conclusions

Pheochromocytoma is a rare yet clinically significant disease that affects the lives of many people. Even in subspecialty settings, incidental findings can help patients address underlying medical pathologies they were not aware of. Findings associated with the disease, such as neurofibromatosis and adrenal masses, must be quickly identified and passed on to the relevant professional to coordinate confirmatory diagnostic care and surgical consultation for the patient. CT findings are a common modality by which these diagnoses are preliminarily made, and it’s important to address incidental findings as they arise. Pheochromocytoma may be implicated in the pathophysiology of pain, although it is difficult to determine at this point. Further research is needed on these associations, and thus, when incidental findings arise in the future, research should be continued to strengthen knowledge of this rare potential phenomenon.
